# Structural and Interpersonal Sources of Perceived Social Support for Transgender Older Adults.

**DOI:** 10.1093/geroni/igaf122.1072

**Published:** 2025-12-31

**Authors:** Anyah Prasad, Hailey Jung, Karen Fredriksen-Goldsen

**Affiliations:** Vanderbilt University, Boston, Massachusetts, United States; University of Washington, Seattle, Washington, United States; University of Washington, Seattle, Washington, United States

## Abstract

For minoritized and health disparate transgender older adults, perceived social support is a crucial psychosocial resource for successful aging. People form convoys of social networks to support their needs, but negative environmental context may threaten transgender older adults’ perceived availability of social resources. This study uses data from the Aging with Pride: National Health and Sexuality Gender Study (NHAS) and the Movement Advance Project (MAP) to examine both the interpersonal and structural determinants of transgender older adults’ perceived social support. In 2017, NHAS surveyed 189 transgender older adults on their perceived social support and asked to report the number of close ties in their social networks. For the same year MAP tracked trans supportive and anti-transgender state policies and categorized all the states in the US as high/medium and low/negative equality states. NHAS transgender respondents from 32 states, were on average 67 years old, about a third reported being racial/ethnic minority and about half were below the 200% federal poverty level. About two thirds resided in medium/high equality states and about a third resided in low/negative equality states. In the regression analysis, after accounting for the demographic variables, transgender older adults’ perceived social support increased significantly with every additional close network tie (β = 0.11, p < 0.001). Net of social network size, transgender respondents in high/medium equality states reported feeling significantly more supported than those in low/negative equality states (β = 0.34, p = 0.033). These findings highlight that the new wave of anti-transgender legislations will have a lasting negative impact across transgender individuals’ life course.

